# Comparison of pilocarpine‐ versus exercise‐induced sweat sodium concentration across exercise intensities in trained athletes

**DOI:** 10.14814/phy2.70724

**Published:** 2026-01-14

**Authors:** Christopher T. Harris, Lindsey Hunt, Sam O. Shepherd, Tamara D. Hew‐Butler, Andrew V. Blow

**Affiliations:** ^1^ Precision Fuel & Hydration Christchurch UK; ^2^ School of Medicine Wayne State University Detroit Michigan USA

**Keywords:** endurance, exercise, hydration, pilocarpine, sodium, sweat

## Abstract

Pilocarpine‐induced sweat testing offers a laboratory‐based method for assessing sweat composition, but its comparability to exercise sweating remains unclear. Establishing a relationship between this resting test and exercise sweating is important for practitioners when in‐exercise sampling is impractical. This study compared sweat sodium concentration ([Na^+^]) between pilocarpine‐ and exercise‐induced sweat across exercise intensities. 15 well‐trained athletes (10 male, 5 female) performed 3 × 20 min cycling bouts (low [LO], moderate [MOD], and high [HI] intensity) and 4 pilocarpine sweat tests. Sweat was collected from the forearm using pilocarpine iontophoresis at rest, and a macroduct collector during exercise. Exercise [Na^+^] increased with intensity (LO = 44.5 ± 15.6, MOD = 54.9 ± 16.9, HI = 61.3 ± 21.3 mmol·L^−1^; *p* < 0.001) alongside sweat rate (LO = 0.62 ± 0.2, MOD = 1.26 ± 0.3, HI = 1.92 ± 0.6 L·h^−1^). Pilocarpine [Na^+^] overestimated exercise [Na^+^] at LO, matched at MOD, and underestimated at HI. Pilocarpine [Na+] was stable across four visits (*p* = 0.263, coefficient of variation 5.5%). In trained athletes, pilocarpine testing shows intensity‐dependent agreement with exercise [Na^+^]: closest at moderate workloads, with predictable bias at the extremes. Under standardized conditions, it provides a practical alternative for hydration planning when exercise testing is not feasible.

## 1 INTRODUCTION

Humans possess an exceptional ability to thermoregulate via eccrine sweating, surpassing all other mammals. This evolutionary trait enables us to tolerate and perform in hot environments, particularly during prolonged exercise. However, this capacity also incurs a physiological cost; that is, marked fluid and electrolyte losses that, if unreplenished, can impair performance and health. Even ~1%–2% body mass loss can impair endurance performance (Bardis et al., 2013), a finding replicated under blinded conditions (Adams et al., 2018). Sodium ([Na^+^]), the primary electrolyte lost in sweat, plays a key role in maintaining plasma volume, fluid retention, and neuromuscular function during exercise (Sawka et al., 2007; Shirreffs & Sawka, 2011). Other conditions such as muscle cramps (Stofan et al., 2005) and hyponatremia (Lewis et al., 2018) have been attributed to excessive sweat [Na^+^] losses during exercise.

Substantial inter‐individual variability exists in both sweat rate and sweat [Na^+^] (Baker, 2017), although variations in sweat [Na^+^] are largely influenced by genetic factors affecting cystic fibrosis transmembrane conductance regulator (CFTR) function (Eichner, 2008), with smaller effects from diet (reviewed by Baker, 2019) and heat acclimation status (Kirby & Convertino, 1986). In response, both sweat sodium and sweat rate testing have become increasingly common in applied sports settings, enabling personalized hydration strategies. Methods to induce sweating include pharmacological stimulation (e.g., pilocarpine iontophoresis), passive heat exposure, and exercise (Baker, 2016). Patch testing during exercise is the most common technique used to collect sweat for subsequent analysis of [Na^+^], and has the advantage of generating information under highly sport‐specific conditions. However, issues with patch contamination (due to incorrect handling) or evaporative loss of sweat from patches can skew results. Pilocarpine iontophoresis is a technique that induces localized sweating via electrical stimulation of cholinergic receptors, and is considered an accurate and reliable, non‐invasive method for measuring sweat [Na^+^]. It is widely used in clinical diagnostics (e.g., cystic fibrosis testing) (Gibson & Cooke, 1959) and has gained popularity in sports science for its ease of use under resting conditions, meaning testing can be conducted in any environment without impacting an athlete's training requirements. However, the extent to which pilocarpine‐induced sweat [Na^+^] reflects that of exercise‐induced sweat [Na^+^] remains unclear.

Pilocarpine‐stimulated local sweat rates correlate with whole‐body exercise sweat rates, but differences emerge at higher sweat outputs (e.g. >1.5 L·h^−1^; Buono et al., 1991; Vimieiro‐Gomes et al., 2005). Furthermore, previous work has demonstrated a positive linear relationship between sweat rate and sweat [Na^+^] (Buono et al., 2008), although these findings were largely based on untrained individuals with sweat rates <1 L·h^−1^. However, the majority of endurance athletes exhibit sweat rates >1.2 L·h^−1^ in training and competition (Barnes et al., 2019). Thus, it is critical to understand at what intensity (and/or sweat rate) the sodium replacement advice generated through pilocarpine‐induced sweat [Na^+^] testing is relevant, to ensure the correct guidance for sodium intake during exercise is provided to athletes. Therefore, the aim of the present study was to determine the agreement between pilocarpine‐induced sweat [Na^+^] and exercise‐induced sweat [Na^+^] across various exercise intensities (low, moderate, and high), which drive different rates of sweat loss.

## 2 MATERIALS AND METHODS

### 2.1 Participants

Fifteen well‐trained (as defined by De Pauw et al., 2013) cyclists/triathletes (10 male, 5 female) volunteered for the study and met the inclusion criteria (anaerobic threshold >3.5 W·kg^−1^ for females; >4 W·kg^−1^ for males; >10 h weeks training volume for at least 12 weeks). Mean (±SD) age, weight, average 12 week training volume, and anaerobic threshold were: 35 ± 8 years, 66.1 ± 8.3 kg, 12.1 ± 1.7 h, and 4.3 ± 0.3 W·kg^−1^ and 3.7 ± 0.4 W·kg^−1^ for males and females, respectively. All female participants completed a menstrual cycle history questionnaire that assessed typical cycle length, variability over the preceding 3 months, bleeding duration and contraceptive use. Eumenorrhea was defined as regular, spontaneous menstrual cycles occurring every 26–35 days with <7 days of cycle‐to‐cycle variability and no current or recent hormonal contraception use. Participants who did not meet these criteria were excluded. All testing was completed during the self‐reported early follicular phase (days 1–7), counted from the first day of menstrual bleeding. No restrictions were imposed on participants' dietary intake. Urine specific gravity (USG) was measured prior to each trial to ensure adequate pre‐test hydration (USG 1.010–1.025; Armstrong et al., 2025). The study was approved by the Wayne State University Institutional Review Board and written informed consent was obtained from each subject prior to participation in the study.

### 2.2 Experimental design

Four laboratory visits were scheduled at a consistent time of day, >120 min postprandial, and spaced weekly. Males completed the visits over 4 weeks; females over 6 weeks, to align with the self‐reported early follicular phase of their menstrual cycle. Trials were conducted at room temperature (~20°C, ~35% humidity). Temperature and humidity were monitored prior to and throughout each trial, with ambient conditions maintained by adjusting external airflow (e.g. opening and closing windows) and using a commercial dehumidifier (Igenix IG9851, Norfolk, UK). External airflow (~4 m·s^−1^) was also directed at the face and torso.

#### 2.2.1 Pilocarpine sweat test

Sweat [Na^+^] concentration was measured at rest at the beginning of each trial. Local sweating was induced on the sterile left ipsilateral forearm via two stainless‐steel electrodes and two iontophoretic discs (solid agar gel of 96% water, 0.5% pilocarpine nitrate; Webster Sweat Inducer, Wescor Inc., Utah, USA, Catalogue no. SS‐032) through which a 1.5 mA iontophoretic current was applied for ~5 min. Once stimulated, participants rested for a further 15–30 min to collect ~85‐μL sweat using a Wescor sweat collection capsule (Wescor Environmental, Utah, USA) via hydraulic pressure. Samples were then measured for sodium concentration by passing through a conductivity cell (Sweat Chek™, Wescor Inc.). Local sweat rates were not quantified during pilocarpine collections. Because pilocarpine evokes localized sweating at rest (independent of whole body thermoregulatory drive), the resulting [Na^+^] primarily reflects local glandular function and local sweat flow; thus, it is not directly comparable to whole‐body sweating rates measured during exercise. Repeatability of the pilocarpine sweat test was assessed using the coefficient of variation (CV) across the four repeated measures in each participant (*n* = 15).

#### 2.2.2 Visit 1: ramp test and threshold determination

Participants performed a standardized warm‐up (2 min easy effort building cadence to 90+ rpm, 3 min moderate effort at 90 rpm, 4 × [10 s >105 rpm, 20s easy effort], 3 min easy effort) followed by an incremental ramp test to determine peak power output. Anaerobic threshold power was estimated using the final 1 min mean power multiplied by 0.75. The workload targets for the different conditions–50% (LO), 75% (MOD), and 100% (HI)–were then calculated for each individual based on their estimated anaerobic threshold power, which was then multiplied by 0.5, 0.75, and 1.

#### 2.2.3 Visits 2–4: experimental trials

Following the same standardized warm‐up as described above, participants completed a 20 min interval at a LO, MOD, or HI intensity in a randomized order. The Wescor sweat collection capsules were applied to the left ipsilateral forearm 5 min into each trial to avoid contamination of residual sodium in the sweat glands at the start of sweating. Collection capsules were left on until completion of the 20 min. Heart rate (HR; Wahoo Tickr, Wahoo Fitness, Atlanta, USA) and ratings of perceived exertion (RPE; Borg, 1998) were recorded at 5, 10, 15, and 20 min of each trial.

Participants' voided immediately before exercise; no fluids were ingested during trials and no urine was passed until post‐weighing. Sweat rate (L·h^−1^) = (pre‐ to post‐exercise mass change + fluid intake − urine output)/exercise duration. Nude body mass (NBM; kg) was measured to the nearest 0.1 kg at the same time as USG measurements. All cycling tests were performed on a magnetic resistance ergometer (Wattbike Atom; Wattbike Ltd., Nottingham, UK).

### 2.3 Statistical analysis

All data were analyzed using the Statistical Package for Social Sciences (SPSS Statistics, version 26, IBM Corporation, Amonk, New York, USA). Normality was confirmed via Shapiro–Wilk tests. A two‐way repeated measures ANOVA with two levels for sweat collection method (pilocarpine vs. exercise induced) and three steps for exercise intensity (LO, MOD, HI). One‐way repeated measures ANOVAs evaluated intra‐individual variability across four visits and compared sweat [Na^+^] across three exercise intensities. Post‐hoc comparisons used Bonferroni correction. Agreement between pilocarpine and exercise‐induced sweat [Na^+^] concentrations was assessed using a two‐way mixed‐effects Intraclass Correlation Coefficient (ICC) model with absolute agreement. All data are reported as mean ± SD unless otherwise reported. Statistical significance was set at *p* < 0.05.

## 3 RESULTS

Summary data for power output, heart rate, cadence and rating of perceived exertion across three exercise trials are presented in Table 1. Pre‐exercise USG confirmed all participants arrived well hydrated to all visits (1.017 ± 0.004 [1.010–1.025]), indicating consistently euhydrated states.

**TABLE 1:** Values for mean ± SD [min–max] power, heart rate, cadence and RPE during LO, MOD, and HI intensity exercise trials.

Condition	Power (W·kg^−1^)	Heart rate (beats·min^−1^)	Cadence (revolutions·min^−1^)	BORG rating of perceived exertion (AU)
LO	2.1 ± 0.3 [1.6–2.6]	115 ± 8 [101–130]	81 ± 2 [79–85]	6.8 ± 0.7 [6–8]
MOD	3.1 ± 0.4 [2.4–3.9]	144 ± 10 [124–159]	84 ± 4 [82–91]	12.1 ± 1.3 [10–15]
HI	4.2 ± 0.5 [3.5–5.1]	168 ± 10 [144–183]	86 ± 4 [83–91]	18 ± 1.4 [16–20]

Abbreviation: AU, arbitrary units.

**FIGURE 1:**
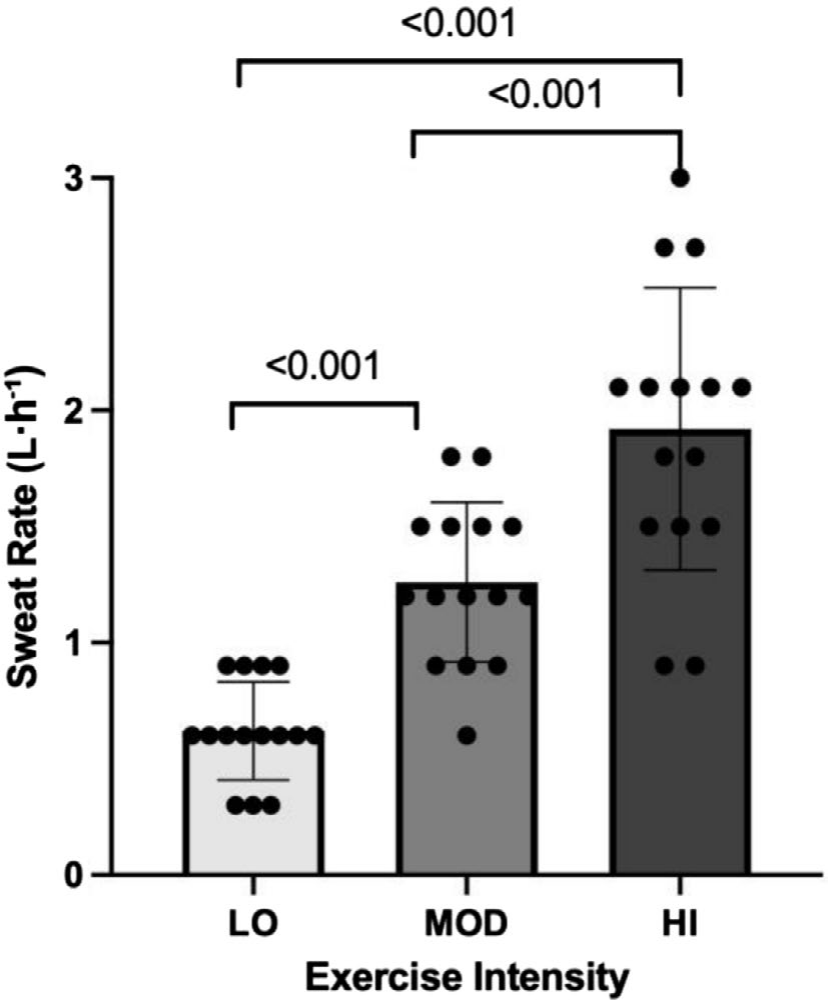
Exercise‐induced sweat rate as a function of exercise intensity. Bars represent mean ± SD.

A one‐way repeated measures ANOVA revealed a significant main effect of exercise intensity on sweat rate (*p* < 0.001, *η* ^*2*^ = 0.785; Figure 1). Post‐hoc comparisons indicated that sweat rate increased significantly across all intensity levels (*p* < 0.001) following a hierarchical pattern: HI > MOD > LO.

**FIGURE 2:**
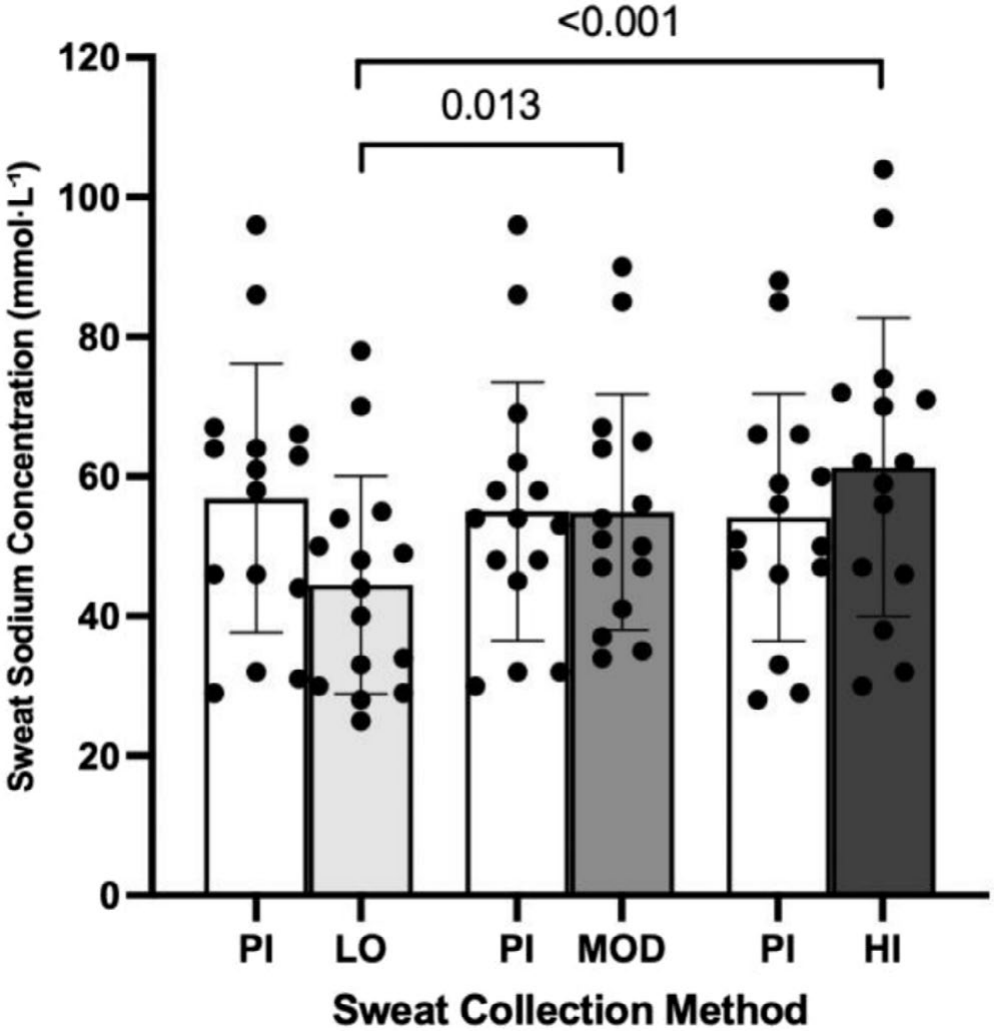
Comparison of pilocarpine (PI) and exercise‐induced sweat sodium concentrations across LO, MOD, and HI intensity exercise. Bars represent mean ± SD.

When comparing pilocarpine and exercise‐induced sweat sodium concentrations no main effect of sweat collection method was observed (*p* = 0.163, *η* ^*2*^ = 0.134). There was, however, a significant main effect of exercise intensity (*p* < 0.001, *η* ^*2*^ = 0.545) on sweat sodium concentration during exercise. A significant interaction effect of sweat collection method (pilocarpine vs. exercise induced) × exercise intensity was also observed (*p* < 0.001, *η* ^*2*^ = 0.732). Post‐hoc analysis revealed significant increases in exercise sweat [Na^+^] between LO and MOD (*p* = 0.013) and between LO and HI (*p* < 0.001), with no significant difference between MOD and HI (*p* = 0.07; Figure 2).

**FIGURE 3:**
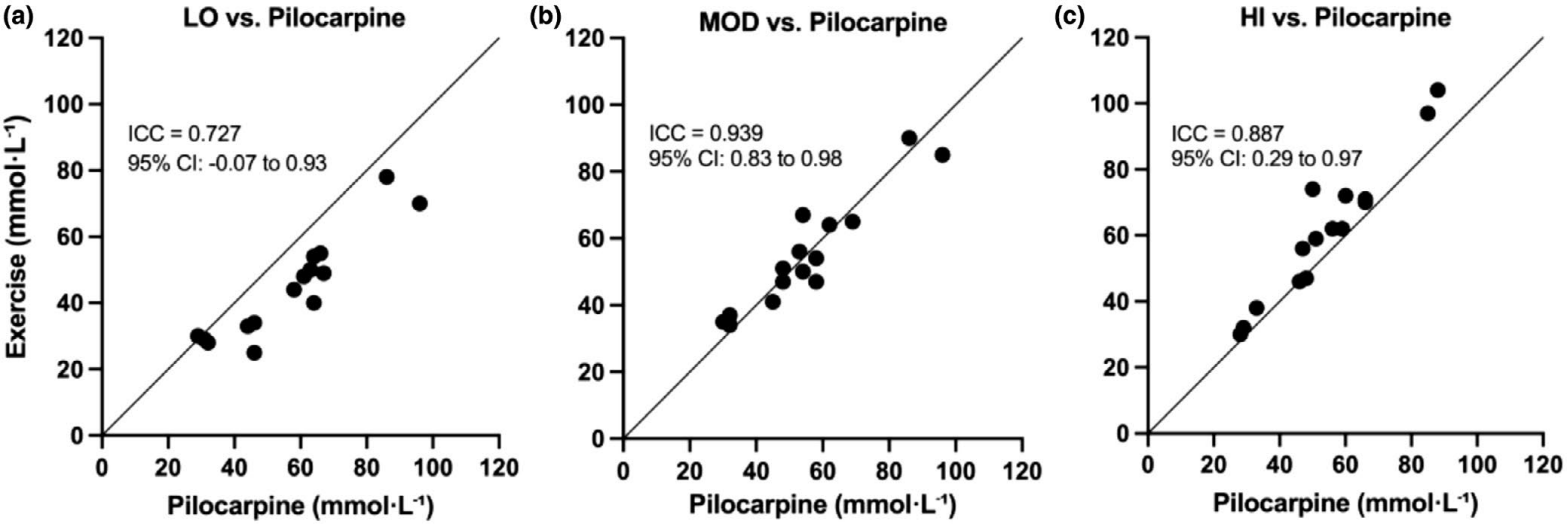
Agreement between individual pilocarpine‐ and exercise‐induced sweat [Na^+^] across LOW (a), MOD (b) and HI (c) exercise intensities. The line of identity (*y* = *x*) is shown for reference.

Intraclass correlation coefficients (ICCs) were calculated to assess the level of agreement between pilocarpine‐ and exercise‐derived sweat sodium concentrations at each exercise intensity. Moderate agreement was observed at low intensity (ICC = 0.727, 95% CI: −0.073 to 0.931, *p* < 0.001), while agreement was excellent at moderate intensity (ICC = 0.939, 95% CI: 0.829–0.979, *p* < 0.001) and good‐to‐excellent at high intensity (ICC = 0.887, 95% CI: 0.293–0.971, *p* < 0.001; Figure 3a–c).

Across four repeated pilocarpine test results the mean results were Visit 1: 55 ± 18 [30–89], Visit 2: 57 ± 19 [29–96], Visit 3: 55 ± 19 [30–96], Visit 4: 54 ± 18 [28–88]. The mean CV was 5.5 ± 3%, indicating low within‐subject variability. Additionally, the intraclass correlation coefficient (ICC) was 0.94 (95% CI: 0.88–0.98), confirming high test–retest reliability.

## 4 DISCUSSION

The present study compared pilocarpine‐ and exercise‐induced sweat sodium concentrations ([Na^+^]) across low, moderate, and high exercise intensities in trained endurance athletes. Our main findings were that: (1) exercise sweat [Na^+^] increased progressively with intensity, consistent with rising sweat rates; and (2) there was good‐to‐excellent agreement between pilocarpine‐derived sweat [Na^+^] and exercise‐induced sweat [Na^+^] at moderate and high intensity exercise.

A key finding of the present study was that agreement between pilocarpine‐ and exercise‐derived [Na^+^] was strongest at moderate workloads. In addition, ICC analysis also demonstrated good‐to‐excellent agreement between pilocarpine‐ and exercise‐derived [Na^+^] during high intensity exercise. During exercise, sweat output is driven by the evaporation requirement for heat balance (Gagnon et al., 2013), whereas pilocarpine bypasses thermoregulatory control and directly activates muscarinic receptors to induce local sweat production (LeGrys et al., 2009). Pilocarpine typically elicits moderate‐to‐high local sweat rates (∼0.4 mg·cm^−2^·min^−1^) (LeGrys et al., 2009; Vimieiro‐Gomes et al., 2005), and previous work shows good agreement between pilocarpine‐evoked local sweat rate and exercise sweat rate at moderate outputs, though disparities emerge at higher outputs (Vimieiro‐Gomes et al., 2005). This suggests that sweat rates observed during moderate and high intensity exercise (between ~1 and ~3L·h^−1^) in the present study likely represent a range where there is a reasonable balance between glandular secretion and ductal reabsorptive efficiency. Under these conditions, pilocarpine iontophoresis may provide a useful estimate of sweat [Na^+^] that could help guide sodium replacement during moderate and high‐intensity exercise. Future research examining whether pilocarpine‐derived [Na^+^] values correspond with international hydration markers, such as serum sodium concentration and blood plasma volume, would further strengthen the case for its use in applied settings.

The observation of an intensity‐dependent rise in exercise sweat [Na^+^] with increasing intensity likely reflects the physiology of eccrine sweat gland function. Sodium reabsorption occurs via epithelial sodium channels (ENaCs) and Na^+^/K^+^‐ATPase pumps in the reabsorptive duct. These transporters actively reclaim sodium ions from the primary sweat as it travels toward the skin surface (Reddy & Quinton, 1994; Sato, 1977). At high sweat rates, shortened transit time through the duct limits the opportunity for complete sodium reabsorption, elevating sweat [Na^+^]. In contrast, lower sweat rates allow longer ductal residence time, facilitating sodium reabsorption and thus lower [Na^+^] concentrations in sweat (Buono et al., 2008; Patterson et al., 2000).

One practical advantage of pilocarpine testing over exercise‐based patch collection lies in its relative consistency. Exercise sweat measurements are subject to fluctuations in external conditions (temperature, humidity), hydration state, body mass shifts, and inter‐trial variability in exercise effort. In contrast, pilocarpine testing under standardized resting conditions reduces many such confounders. In our study, pilocarpine [Na^+^] showed a mean coefficient of variation of ~5.5% across four visits, indicating stable within‐subject variability. Comparable work on other electrolytes (notably potassium) has demonstrated lower day‐to‐day variability in pilocarpine‐induced versus exercise‐associated sweat (Vairo et al., 2017). These findings suggest that the pilocarpine iontophoresis performance under standardized conditions may offer a practical and reproducible method for estimating sweat [Na^+^] in trained athletes, particularly when exercise testing is not feasible. Further research is needed to determine whether pilocarpine‐derived values can reliably inform individualized sodium replacement/hydration strategies across diverse exercise contexts (e.g., middle‐full distance triathlon, marathon, endurance cycling and ultra‐running).

A methodological strength of this study was the consistent use of the forearm as the site for sweat collection in both pilocarpine and exercise conditions. While regional variability in sweat [Na^+^] is acknowledged, the forearm is one of the most representative anatomical sites when compared to whole‐body sodium losses (Baker, 2016; Baker et al., 2009). This makes it a suitable and validated site for localized sweat collection when attempting to understand whole‐body sweat [Na^+^] losses in more practical scenarios, particularly as whole‐body methods are often only available in research settings. Consistent use of the same anatomical region across conditions also reduces potential bias from regional gland and sweat rate differences (Smith & Havenith, 2011) and supports valid within‐subject comparisons (Baker et al., 2016).

## 5 LIMITATIONS

This study used short, temperate condition exercise bouts (20 min; ~20°C; ~35% RH), so extrapolation to longer sessions or hotter/humid conditions also warrants confirmation. Dietary sodium and recent training were not rigidly standardized, but the good‐to‐excellent agreement at moderate and high intensity exercise between pilocarpine and exercise‐induced sweat [Na^+^] suggests high ecological validity of the study outcomes. Whether training status influences the agreement between at‐rest pilocarpine‐induced sweat [Na^+^] and exercise‐induced sweat [Na^+^] remains to be tested. Therefore, caution should be applied when applying these results to untrained or clinical populations. It also remains unclear whether the agreement between pilocarpine‐ and exercise‐derived sweat [Na^+^] differs by sex. Although female participants in the present study were tested during the early follicular phase to minimize hormonal variability, the study was not powered to detect sex‐specific differences, so future work should aim to examine this.

## 6 CONCLUSION

This is, to our knowledge, the first study in trained endurance athletes to quantify how agreement between pilocarpine and exercise [Na^+^] shifts with intensity, at sweat rates up to ~3 L·h^−1^. Practically, a single pilocarpine test obtained under standardized conditions appears fit‐for‐purpose for day‐to‐day hydration strategy planning during moderate‐ and high‐intensity training. The low intra‐individual variability and ease of use make pilocarpine iontophoresis a viable option from which to develop athlete hydration strategies, especially when exercise‐based testing is not feasible.

## AUTHOR CONTRIBUTIONS

Christopher T. Harris, Lindsey Hunt, Sam O. Shepherd, and Andrew V. Blow. Blow are all employees of Precision Fuel & Hydration (PF&H). Financial support for this research was provided by PF&H, a division of Precision Hydration Ltd. in the form of salaries. The views expressed in this article are those of the authors and do not necessarily reflect the position or policy of Precision Hydration Ltd. Wescor is the manufacturer of the Sweat Chek device used in the study to measure sweat sodium concentration, but has no commercial or financial relationship with Precision Hydration Ltd. beyond use of commercially available equipment.

## ETHICS STATEMENT

This study was approved by the Wayne State University Institutional Review Board.

## Data Availability

The data that support the findings of this study are available from the corresponding author upon reasonable request.
